# Prevalence and impact of potentially inappropriate medication on community-dwelling older adults

**DOI:** 10.7705/biomedica.5787

**Published:** 2020-10-20

**Authors:** Alejandra Fernández, Fernando Gómez, Carmen-Lucía Curcio, Edison Pineda, Juliana Fernandes de Souza

**Affiliations:** 1 Facultad de Ciencias para la Salud, Universidad de Caldas, Manizales, Colombia; 2 Departmento de Fisioterapia, Universidad Federal de Rio Grande do Norte, Natal, Brasil

**Keywords:** Potentially inappropriate medication list, aged, comorbidity, frailty, lista de medicamentos potencialmente inapropiados, anciano, comorbilidad, fragilidad

## Abstract

**Introduction:**

Potentially inappropriate medication is associated with adverse health and functional outcomes, as well as increased health care costs.

**Objective:**

To estimate the prevalence and types of potentially inappropriate medication according to the Beers criteria in community-dwelling older persons and to identify the major clinical and functional consequences of potentially inappropriate medication during two years of following.

**Materials and methods:**

We conducted a longitudinal, descriptive, and observational study that included 400 65-year or older community-dwelling people (48% women) selected by simple random sampling in 2012. In 2014, 372 people were re-evaluated and classified into two groups based on the presence or absence of potentially inappropriate medication through the follow-up period.

**Results:**

In total, 31% had polypharmacy (5-9 medications) and 1,8% had excessive polypharmacy (10 or more medications). The mean of the number of medications was higher in the potentially inappropriate medication group (3 vs. 5.78; p<0.001) and 21.9% still had the potentially inappropriate medication status during the follow-up; of them, 75% had one potentially inappropriate medication and 23% two. The presence of potentially inappropriate medication was more frequent among frail and depressed male individuals with a bad health self-assessment and comorbidities, especially diabetes mellitus and chronic obstructive pulmonary disease. In the group with sustained potentially inappropriate medication, we found a worsening health self-assessment, increased frailty, a higher incidence of recurrent falls and prevalence of depression, as well as a higher hospital admission rate, ambulatory medical consultation, and more prescribed medications. We did not find an impact on functional capacity.

**Conclusions:**

We validated the negative effects of potentially inappropriate medication in the long run for the health of older people and, therefore, potentially inappropriate medications should be monitored in primary care services to avoid greater risks.

## Abstract (es)

**Introducción:**

La medicación potencialmente inapropiada se asocia con consecuencias clínicas, geriátricas, funcionales y farmacoeconómicas negativas.

**Objetivo:**

Estimar la prevalencia y los tipos de medicación potencialmente inapropiada según los criterios de Beers en ancianos que viven en comunidad y determinar las principales consecuencias clínicas y funcionales a lo largo de dos años de seguimiento.

**Materiales y métodos:**

Se hizo un estudio descriptivo, observacional y longitudinal que incluyó 400 mayores de 65 años (48% mujeres) residentes en la comunidad seleccionados mediante muestreo aleatorio simple en el 2012. En el seguimiento del 2014 se reevaluaron 372 de ellos y se clasificaron en dos grupos: quienes a lo largo de los dos años siguieron tomando medicación potencialmente inapropiada y quienes no.

**Resultados:**

El 31% de los ancianos estaban polimedicados, (5 a 9 medicamentos) y 1,8% polimedicados de forma excesiva (10 o más medicamentos). El promedio de consumo de medicamentos era mayor en el grupo de medicación potencialmente inapropiada (3 *Vs.* 5,78; p<0,001), y el 21,9% siguieron recibiendo medicación poitencialmente inapropiada durante el seguimiento; de ellos, el 75% recibía un medicamento de este tipo y el 23%, dos. El uso de dicha medicación fue más frecuente en hombres frágiles con una mala percepción de la propia salud, depresión y un mayor número de comorbilidades, especialmente diabetes mellitus y enfermedad-pulmonar-obstructiva-crónica. En el grupo que siguió recibiendo medicación potencialmente inapropiada la percepción de la propia salud empeoró, con un incremento de la fragilidad, las caídas recurrentes y la depresión, así como en los ingresos hospitalarios y las consultas médicas y mayor cantidad de medicamentos formulados. No se evidenció un impacto en la capacidad funcional.

**Conclusiones:**

Se corroboran los efectos negativos a largo plazo de la medicación potencialmente inapropiada en la salud de los ancianos, por lo cual se la debe monitorizar en la atención primaria para evitar mayores riesgos.

The accelerated increase in the proportion of older people implies greater challenges for modern geriatrics (1). Up to 80% of people older than 65 have at least one chronic health condition (2). Usually, these patients are more vulnerable to worse outpatient care quality, especially regarding the prescription of medications (3). There is a proportional relation between age and the number of prescribed medications, as well as with the development of problems related to medication (4).

Among these problems related to medication, polymedication or polypharmacy is quantitatively determined according to the number of medications, and from a qualitative viewpoint, by the use of inadequate medications (5). From a quantitative point of view, polymedication is defined as the prescription of five or more medications per day, and excessive polymedication as the consumption of ten or more medications per day (6). Qualitatively, polymedication is determined by the presence of more risks than benefits when administering medication given the characteristics of the drug and those of the patient, and it is better known as potentially inappropriate medication (7). Potentially inappropriate medications are those medications whose individual or combined administration should be avoided in older people because they may cause more harm than benefits *vis a vis* safer alternatives (8).

Explicit and implicit assessment strategies have been developed to detect potentially inappropriate medications. Implicit strategies are based on judgment and require clinical information to be interpreted and assessed, as opposed to explicit strategies that are supported in predesigned lists for the elderly population to determine which medication is more beneficial than harmful (9). In outpatient senior citizens, AGS Beers’ criteria are more efficient for detecting potentially inappropriate medications (10,11). Polymedication and the use of inappropriate drugs have direct consequences on adverse effects, interactions, frequency of consultation in health services, and functional outcomes; they are also associated with comorbidity, syndromes, and geriatric conditions, and they increase the risk of hospitalization influencing morbidity, mortality, and the quality of life of senior patients (12).

The objectives of this study were to estimate the prevalence and types of potentially inappropriate medications according to Beers’ criteria in people older than 65 living in the community and to describe the main consequences of potentially inappropriate medications regarding the clinical, functional, syndromic, and geriatric conditions, as well as the use of health services and the mental wellbeing during a two-year follow-up (2012-2014).

## Materials and methods

### Study population

We conducted an analysis of the data obtained from the International Mobility in Aging Study (IMIAS), a longitudinal population study among 1,995 people between 65 and 74 years of age living in the community from five different cultural and social contexts: Kingston (Ontario, Canada), Saint-Hyacinthe (Quebec, Canada), Tirana (Albany), Manizales (Colombia), and Natal (Brazil). The objective of the IMIAS was to understand how factors throughout the course of life affect mobility in old people. Its characteristics and details have been described elsewhere (13).

**Figure 1:**
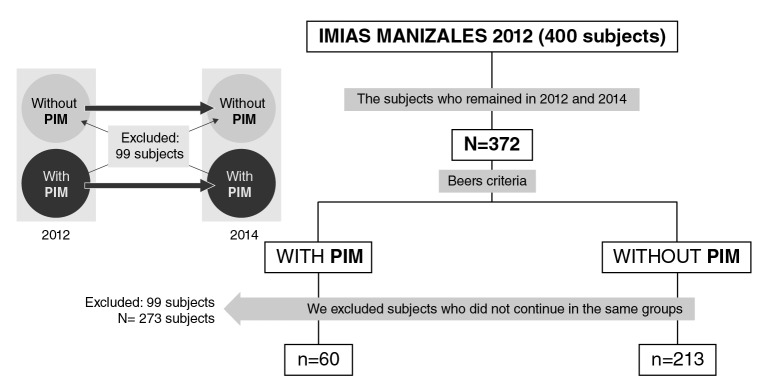
Sample selection process

In the present study, we used the data of the population from Manizales (Colombia): 200 males and 200 females in 2012, and 372 senior citizens followed up until 2014 (7% attrition). The sample selection procedure is shown (figure 1). After classifying the participants with potentially inappropriate medications in 2012 according to Beers’ criteria, we followed them up until 2014 and excluded those who did not comply with this condition: 60 participants continued to take potentially inappropriate medications and 213 did not, but they were included in the longitudinal analysis.

### Data collection

The information was obtained during house visits from structured interviews carried out by previously trained staff. The data regarding medications were obtained directly from the patients’ prescriptions and from the packaging of drugs prescribed by a doctor or self-prescribed by the patient taken during the previous 15 days. All medications were identified using the Anatomical Therapeutic Chemical (ATC) Classification suggested by the World Health Organization (WHO) (14).

### Main measurement

For potentially inappropriate medication, we used Beers’ criteria, an explicit description strategy developed in 1991 regularly updated by the American Geriatrics Society (AGS) according to new findings recorded in the literature. These have been validated in several media and has a better performance for the detection of potentially inappropriate medications in people living in communities compared with other strategies (15). We used version 2 – 2015 (tables 2, 3, 4) and we took into account those medications classified as inappropriate (16,17).

**Table 2:** Prevalence ratio for various consequences among the subjects with potentially inappropriate medications, 2012-2014 (n=60)

**Characteristic**	**Significance (p<0.05)**	**PR (CI)**
Self-rated health	p<0.001	2.93 (1.62-5.27)
Number of chronic diseases (two or fewer vs. three or more)	p<0.001	2.50 (1.34-4.69)
Diabetes mellitus	p<0.001	4.38 (2.11-9.04)
Fragility	0.004	4.06 (1.60-10.28)
Recurring falls	0.028	2.43 (1.08-5.84)
Depression, CES-D scale >16 points	p<0.001	3.28 (1.70-6.33)
Hospitalizations in the last two years	p<0.001	2.45 (1.34-4.50)
Number of hospitalizations in the last two years	0.010	3.75 (0.82-17.24)
Number of doctor consultations in the last year	0.001	4.22 (1.73-10.29)
Total prescribed medications (Polymedication vs. no polymedication)	p<0.001	8.93 (4.67-17.08)

*PR (CI): Prevalence ratios (confidence interval); CI=95%

### Covariables

*Sociodemographic variables.* We included questions about general demographic aspects, such as age (years); sex (male/female); educational level (number of complete formal education years: 0-5, 6-11, or 12 or more); marital status (single, married, widow/widower, or separated); income (current legal minimum wage at the moment of the interview divided into two groups: less than USD$ 300 or more than USD$ 300 per month).

*Self-rated health.* It was assessed using the question “Do you consider your health to be: good, very good, regular, bad, very bad” (18); for the analysis, it was dichotomized between good and very good vs. regular, bad, and very bad.

*Presence of chronic illnesses*. Participants were asked if a physician had ever told them they had one of the following chronic illnesses: high blood pressure, osteoarthrosis/arthritis, heart disease, chronic obstructive pulmonary disease, diabetes, cancer, or osteoporosis. Additionally, we obtained the number of self-reported chronic conditions (19).

*Medications.* We recorded the total number of medications or active ingredients a person consumed regularly for at least two weeks with a specific objective either prescribed by health personnel or self-prescribed. They were recorded according to the ATC classification suggested by WHO (20). Medication packages and their presentations were checked. Duplicate medications were not taken into account for the analysis. According to their quantity they were classified into no polymedication (0-4 medications), polymedication (5-9 medications), and excessive polymedication (10 or more medications) (3).

*Visits to the doctor*. We registered the number of outpatient visits to the doctor in the last year as referred by the participant.

*Hospitalization*. We recorded the number of times the person was hospitalized in the previous two years. This variable was analyzed only during the follow-up (2014).

*Geriatric conditions*. We took into account the fragility, as defined by Fried, *et al.* (21) and documented in previous publications of the IMIAS study (22).

*Recurring falls.* This variable referred to the self-report of two or more falls in the previous year (23,24).

*Depression*. We used the depression scale of the *Centro de Estudios Epidemiológicos* (CES-D) (25-27), which has 20 items related to depressive symptoms and rated from 0 to 60 points where scores >16 are considered suggestive of depression (28).

*Cognitive impairment*. We applied the Leganés cognitive test, which evaluates six cognitive areas: High scores correspond to an adequate cognitive functioning and >22 suggest cognitive impairment (28).

*Activities of daily living.* We asked the participants the following question: “Do you have any difficulty to independently perform one of the following activities: walking in a room, getting dressed, bathing, eating, climbing stairs, climbing into bed, and going to the toilet” (29,30) and then classified them into two groups: those who had no difficulties for performing the activities vs. the rest.

All the procedures were endorsed by the University of Caldas Bioethics Committee.

### Statistical analysis

We carried out a descriptive analysis (frequency distribution, averages, and standard deviation). We did a bivariate analysis using the Student’s t test for continuous variables and the chi-squared and Fisher’s exact tests for categorical variables. The normality of the continuous variables was evaluated using the Kolmogorov-Smirnov test. We used generalized linear models with robust Poisson regression models to estimate prevalence ratios. Multivariate logistic regression was used to identify the correlations of taking potentially inappropriate medications after the adjustment of the demographic, health, and medication variables. We considered p<0.05 as statistically significant. The data were analyzed using SPSS™, version 22.0.

### Ethical considerations

The study was approved by the ethics committee of the *Universidad de Caldas*. All participants signed the informed consent. According to Resolution 8430/1993 from the Colombian Ministry of Health on studies with human beings, it was considered of minimal risk given that participants had a very low probability of suffering any harm as a consequence of the study.

## Results

Table 1 shows the characteristics of the sample at the beginning of the study (2012) and during follow-up (2014) according to participants’ sociodemographic and clinical variables and their geriatric conditions. There were significant differences regarding gender: 66.7% of the total population with potentially inappropriate medications were males. Regarding the clinical status, self-rated health was significantly better (p=0.011) among those not taking potentially inappropriate medications. As regards comorbidity, participants with potentially inappropriate medications reported an average of 2.02 chronic illnesses while those without potentially inappropriate medications only reported 1.31 (p=0.000).

**Table 1:** Characteristics of the population under study, 2012 and 2014

**Characteristics**	**2012**				**2014**			
	**Total** **n=273**	**PIM (no)** **n=213**	**PIM (yes)** **n=60**	**p** **value**	**Total** **n=273**	**PIM (no)** **n=213**	**PIM (yes) 1** **n= 60**	**p value**
Sociodemographic								
Age, average (SD)	69.3 (2.96)	69.1 (2.89)	69.9 (4.15)	0.075	71.4 (3.00)	71.2(2.95)	72.3 (3.146)	0.081
Gender, n (%)				0.010				0.010
Female	131 (48.0)	111 (52.1)	20 (33.3)		131(48.0)	111 (52.1)	20 (33.3)	
Male	142 (52.0)	102 (47.9)	40 (66.7)		142 (52.0)	40(47.9)	40(66.7)	
Marital status, n (%)				0.613				0.389
Single	37 (13.6)	30 (14.1)	7(11.7)		40 (14.7)	34 (16.0)	6 (10.0)	
Married	127 (53.8)	111 (52.1)	36 (60.0)		147 (53.8)	109 (51.2)	38 (63.3)	
Widow/Widower	60 (22.0)	50 (23.5)	10 (16.7)		63 (23.1)	51 (23.9)	12 (20.0)	
Separated	29 (10.6)	22 (10.3)	7 (11.7)		23 (8.4)	19 (8.9)	4 (6.7)	
Education				0.838				0.700
0-5 years n (%)	180 (65.9)	139 (65.3)	41 (68.3)		167 (64.8)	137 (64.3)	40 (66.6)	
6-11 years	43(15.8)	35 (16.4)	8 (13.3)		59 (21.6)	47 (22.1)	12 (20.0)	
12 years or more	50 (18.3)	39 (18.9)	11 (18.3)		37 (13.6)	29 (13.6)	8 (13.0)	
Income, n (%)				0.678				0.609
Less than USD$ 300	219 (80.2)	172 (80.8)	47 (78.3)		167 (61.2)	132 (62.0)	35 (58.3)	
More than USD$ 300	54 (19.8)	41 (19.2)	13 (21.7)		106 (38.8)	81 (38.0)	25 (41.7)	
Clinical characteristics								
Self-rated health				0.011				<0.001
Excellent/Very good	153 (56.0)	128 (60.1)	25 (41.7)		185 (67.8)	156 (73.2)	29 (48.3)	
Regular/Bad/Very bad	120 (44.0)	85 (39.9)	35 (58.3)		88 (32.2)	57 (26.8)	31 (51.7)	
Chronic illnesses								
Average (SD)	1.47 (1.27)	1.31 (1.14)	2.02 (1.52)	<0.001	1.62 (1.31)	1.47 (1.21)	2.16 (1.50)	<0.001
High blood pressure, n (%)	158 (57.9)	118 (55.4)	40 (66.7)	0.247	162 (59.0)	120 (56.3)	41 (68.3)	0.095
Osteoarthritis, n (%)	75 (27.5)	54 (25.4)	21 (35.0)	0.266	83 (30.4)	60 (28.2)	23 (38.3)	0.131
Heart disease, n (%)	49 (17.9)	35 (16.4)	14 (23.3)	0.219	60 (22)	46 (21.6)	14 (23.3)	0.774
COPD, n (%)	32 (11.7)	20 (9.4)	12 (20.0)	0.024	38(13.9)	25 (11.7)	13 (21.7)	0.050
Diabetes mellitus, n (%)	32 (11.7)	17 (8.0)	15 (25.0)	<0.001	37 (13.6)	19 (8.9)	18 (30.0)	<0.001
Osteoporosis, n (%)	35(12.8)	26 (12.2)	9 (15.0)	0.525	39 (14.3)	30 (14.1)	9 (15.0)	0.858
Cancer, n (%)	12 (4.4)	6 (2.8)	6 (10)	0.017	16 (5.9)	8 (3.8)	8 (13.3)	0.050
Medications								
Total, average (SD)	3.67 (2.66)	3.08 (2.3)	5.78 (2.54)	<0.001	4.01 (3.149)	3.35 (2.80)	6.38 (3.195)	<0.001
Prescribed medications	3.0 (2.41)	2.47 (2.14)	4.88(2.38)	<0.001	3.49 (2.88)	2.87 (2.56)	5.72 (2.90)	<0.001
Self-prescribed medications	0.67 (1.03)	0.60 (0.94)	0.90 (1.27)	0.048	0.47 (0.818)	0.44 (0.797)	0.57 (0.89)	0.295
Visits to the doctor in the last year, average (SD)	4.48 (4.03)	4.0 (3.65)	6.18 (4.80)	<0.001	4.7 (4.34)	4,23 (4,29)	6.35 (4.14)	0.001
Hospitalized in the two last years, n (%)	----	----	----	----	73 (26.7)	48 (22.5)	25 (41.7)	0.003
Number of times, average (SD)	----	----	----	----	1.55 (1.00)	4,23 (4,29)	1.96 (1.27)	0.010
Activities of daily living, without difficulties	212 (77.6)	170 (79)	42 (70)	0.031	200 (78)	165 (77.4)	35 (58.3)	0.042
Activities of daily living, one or more difficulties	61 (22.4)	43 (21)	18 (30)	0.031	73 (22)	48 (22.6)	25 (41.7)	0.042
Geriatric conditions								
Recurring falls, n (%)	45 (16.5)	32(15.0)	13 (21.7)	0.221	29 (10.6)	18 (8,5)	11 (18.3)	0.028
Fragility				0.004				0.003
Vigorous	136(49.8)	112(52.6)	24(40.0)		133 (48.7)	111 (52,1)	22(36.7)	
Pre-fragile	125 (45.8)	96(45.1)	29 (48.3)		120 (44.0)	92 (43.2)	28 (46.7)	
Fragile	12 (4.4)	5(2.3)	7 (11.7)		20 (7.3)	10 (4.7)	10 (16.7)	
Fragile (yes), n (%)	12 (4.4)	5 (2.3)	7 (11.7)	0.002	20 (7.3)	10 (4.7)	10(16.7)	0.002
Depression n (%)	63 (23.1)	43(20.2)	20 (33.3)	0.033	51(18.7)	30 (14.1)	21 (35.0)	<0.001
Cognitive impairment n (%)	13 (4.7)	10 (4.6)	3 (5.9)	0.90	15 (5.5)	11 (5.1)	4 (6.7)	0.46

**Figure 2:**
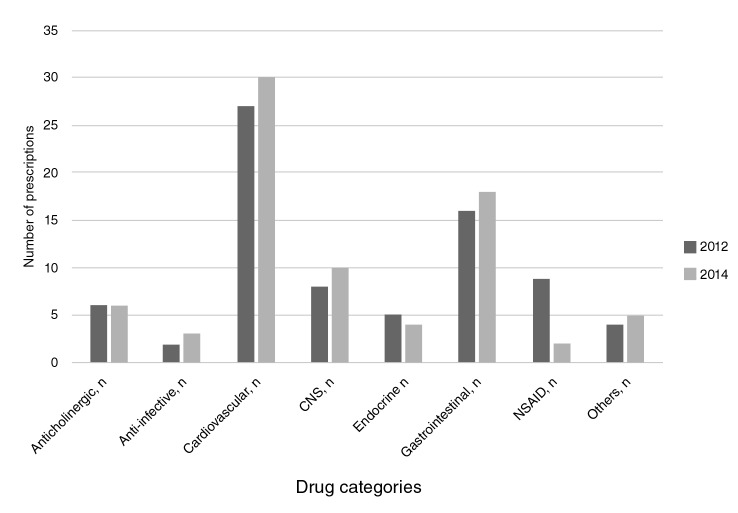
Categories of potentially inappropriate medication prescribed (2012-2014).

The average number of consumed medications was 3.67 (Stangard deviation, SD=2.66); a higher consumption was found among participants taking potentially inappropriate medications than among those who did not (3 vs. 5.78; p<0.001); 31% had polymedication (5-9 medications) and 1.8% had excessive polymedication (10+ medications). At the two-year follow-up, 21.9% of the participants were still taking potentially inappropriate medications of whom, 75% took one, 23% two, and 0.4% three. The use of potentially inappropriate medications was more frequent in males with bad self-rated health, a higher number of comorbidities (especially diabetes mellitus and chronic obstructive pulmonary disease), who were more fragile and had depression. The most frequently taken potentially inappropriate medications were prazosin (alpha blockers) by 33% of participants, proton pump inhibitors by 17%, non-steroidal anti-inflammatory drugs (NSAID) by 12%, and antihistamines by 7.5%, all of which continued to be prescribed two years later (figure 2).

During follow-up, the self-rated health of people taking potentially inappropriate medications was worse and their comorbidities increased, especially diabetes mellitus, as well as their fragility, which increased significantly from 11 to 17% after two years. The number of recurring falls was significantly higher in this group too (18.3% vs. 8.5%). Likewise, depression, the number of hospitalizations and medical consultations, and the total prescribed medications were significantly higher in this group that kept taking potentially inappropriate medications after two years.

Regarding the outcome in both populations after two years, we did not find statistically significant differences among the sociodemographic characteristics. On the other hand, 84.3% of the people with good and very good self-rated health in 2014 belonged to the group not taking potentially inappropriate medications since 2012. From 2012 to 2014 there was an increase in the mean of chronic illnesses, especially in the potentially inappropriate medications group.

The prevalence of diabetes mellitus increased 5% in the population that kept taking potentially inappropriate medications compared to 0.9% of those who did not. Fragility increased significantly in both groups at the two-year follow-up, but it was more significant in the group taking potentially inappropriate medications as 16.7% of those who took them in 2012 were fragile in 2014 compared to 4.7% of those who did not. The percentage of participants with depression was significantly higher in the group taking potentially inappropriate medications. The number of hospitalizations, doctor consultations, and total prescribed medications was higher in the group of patients taking potentially inappropriate medications since 2012.

Table 2 shows the logistic regression analysis used to analyze the associated characteristics in the group of subjects who persisted in taking potentially inappropriate medications during the two years under evaluation. We found an association between potentially inappropriate medications persistence and consequences such as self-rated health, the number of chronic illnesses, especially diabetes mellitus, fragility, recurring falls, depression, and greater use of health care services. We did not evidence a significant impact on functional capacity.

## Discussion

We made a longitudinal analysis of potentially inappropriate medications establishing its prevalence, assessed comorbidity, and functionality outcomes, as well as geriatric syndromes in an elderly population in the community. Quantitative polymedication (five or more medications) was present in 31% of the study population and 21% of them took potentially inappropriate medications, especially heart medication (prazosin), proton pump inhibitors, and NSAID. Potentially inappropriate medications were more frequent in males with bad self-rated health, a higher number of comorbidities, especially diabetes mellitus and chronic obstructive pulmonary disease, who were more fragile and depressed. At the two-year follow-up, potentially inappropriate medications were associated with adverse health outcomes such as worse self-rated health, increased comorbidities, especially diabetes mellitus, increased fragility, recurring falls, and variables associated with adverse health events including hospitalizations, medical consultations, and the total number of prescribed medications.

The prevalence of quantitative polymedication and potentially inappropriate medications was within the ranges reported in the literature: approximately one third of the elderly population living in the community consumes five or more medications (12), and at least one fourth of them took potentially inappropriate medications (31). A Spanish study that included citizens older than 85 living in the community (5) reported a potentially inappropriate medications prevalence of 69% while a Colombian study (11) reported a 6.9% in an outpatient elderly population. A Brazilian longitudinal study on potentially inappropriate medications (32) found a prevalence of 43.8%, which was associated with bad self-rated health, although, contrary to our study, it was more frequent in females.

Both at the beginning of the study and at the follow-up, the most frequently prescribed potentially inappropriate medications were cardiovascular ones, specifically alpha blockers (around 30%), followed by proton pump inhibitors without clear indication, and, lastly, by drugs with a direct effect on the central nervous system, which agrees with findings from other studies (33). More recent studies show a high proportion of hypnotic and anticholinergic medication.

A study in Spanish patients over 85 years old reported high prescription of hypnotics (benzodiazepine-type) and cardiovascular medications such as loop diuretics and NSAID (5). A French study showed that approximately 12% of all potentially inappropriate medications were hypnotics (benzodiazepines) and almost 10%, anticholinergics (tricyclic antidepressants-type) (34). In Taiwan, around 21% of all potentially inappropriate medications were benzodiazepines (35) while in the Brazilian study mentioned previously, methyldopa and clonazepam prescriptions corresponded to 25% of the total prescribed potentially inappropriate medications. In Colombia, the pharmacological group most frequently associated with potentially inappropriate medications was that of NSAID with almost 12% of prescriptions in this age group (11). In our study, proton pump inhibitors without clear indication represented almost 17% of all potentially inappropriate medications prescriptions similar to reports from other countries such as Pakistan, where they accounted for 25% (36).

The number of prescribed potentially inappropriate medications was the same with a small tendency to increase during the two follow-up years, which according to the literature could be associated with an increase in the medication-medication and medication-comorbidity interactions (37). Additionally, in our study, the number of comorbidities during follow-up increased suggesting the need for more medications as evidenced at the two-year mark. This is the tendency reported in longitudinal studies with an increase in comorbidities and, consequently, in polypharmacy and potentially inappropriate medications (23,38).

Diabetes mellitus was associated with the potentially inappropriate medications group during follow-up. It has been previously noted that specific chronic conditions (for instance, mental health disorders or diabetes mellitus) could increase the risk of potentially inappropriate medications since the medications that are usually administered for their control are linked to adverse effects in the elderly population (19). On the other hand, the association between potentially inappropriate medications and depression has been mentioned before (25,38) and it is likely that in our case, they constituted collateral effects of potentially inappropriate medications or medications that promote depressive symptoms (38). Along those lines, the deterioration in self-perceived health during follow-up would respond to the fact that senior patients with the worst self-perceived health think they are sicker and use health care services a lot more (32). Likewise, the association of self-rated health with illnesses, disability, and functional limitations is clear (39).

Regarding the consequences of geriatric conditions and syndromes and potentially inappropriate medications, they have been mostly reported in transversal studies (34). On the other hand, the association of increased fragility in elderly people and potentially inappropriate medications during follow-up was evident, especially those with anticholinergic effects (40). In a transversal study in a French population older than 65 years, a proportional association among fragility, excessive polymedication, and potentially inappropriate medications was found (34); these findings support the recommendation to reduce polypharmacy as part of the interventions to manage fragility. As shown in various studies (41), the role of potentially inappropriate medications in recurring falls due to drowsiness, deterioration of postural reflexes, myorelaxant effects, and extrapyramidal symptoms is clear (24).

As for the use of health care services, we corroborated the association between potentially inappropriate medications and the increase in hospitalization rates, doctor visits (35), and polymedication. In a recent metanalysis of observational studies on potentially inappropriate medications, the authors found that it was significantly associated with emergency room visits, drug adverse reactions, and hospitalizations. Additionally, the Albacete study recently showed that polypharmacy is linked to mortality, incident disability, hospitalization, and visits to the emergency room in fragile and pre-fragile elders. These findings indirectly indicate that physicians are frequently prescribing these medications in this age group, and as such, they can be held responsible for most of the potentially inappropriate medications and the impact they have on people’s health.

Among the strengths of our study, we can mention the documented long-term associations of potentially inappropriate medications with sociodemographic factors, clinical characteristics, and geriatric conditions in a group of elderly citizens in the community. Usually, longitudinal studies are done with quantitative polypharmacy, while this one used qualitative polypharmacy. Another strength is the direct review of patient prescriptions and medication packaging by the health personnel who collected the data, which makes our results more reliable. Finally, we included self-prescribed medications in this category, which is infrequent in potentially inappropriate medications studies.

Our study also had various limitations: Due to selection filters, the sample size might not have been enough to find a significant association among some variables. Another limitation was the narrow age range, 65 to 74 years old, which limits the generalization of results to populations with higher potentially inappropriate medications intake, such as very old or institutionalized people.

In conclusion, polymedication was present in the third part of the elderly population living in the community and the fifth part took potentially inappropriate medications, especially heart and gastrointestinal medications, as well as anti-inflammatories. We corroborated the negative long-term effects of potentially inappropriate medications in the health of the elderly, especially regarding the deterioration of self-rated health, the presence of more comorbidities, like diabetes mellitus and depression, as well as multiple geriatric conditions such as fragility, recurring falls, and increased use of health care services. Therefore, it is important that health care teams constantly monitor these to avoid the risks of polymedication and inappropriate use of medication.
